# Parameterized Hilbert-Type Integral Inequalities in the Whole Plane

**DOI:** 10.1155/2014/169061

**Published:** 2014-08-19

**Authors:** Qiliang Huang, Shanhe Wu, Bicheng Yang

**Affiliations:** ^1^Department of Mathematics, Guangdong University of Education, Guangzhou, Guangdong 510303, China; ^2^Department of Mathematics and Computer Science, Longyan University, Longyan, Fujian 364012, China

## Abstract

By the use of the way of real analysis, we estimate the weight functions and give some new Hilbert-type integral inequalities in the whole plane with nonhomogeneous kernels and multiparameters. The constant factors related to the hypergeometric function and the beta function are proved to be the best possible. We also consider the equivalent forms, the reverses, and some particular cases in the homogeneous kernels.

## 1. Introduction

If *f*(*x*), *g*(*y*) ≥ 0,  0 < ∫_0_^*∞*^ *f* ^2^(*x*)*dx* < *∞*, and 0 < ∫_0_^*∞*^ *g* ^2^(*y*)*dy* < *∞*, then we have (cf. [1])

(1) ∬0∞f(x)g(y)x+ydx dy  <π(∫0∞f2(x)dx∫0∞g2(y)dy)1/2,

where the constant factor *π* is the best possible. Inequality (1) is well known as Hilbert's integral inequality, which is important in analysis and its applications (cf. [1, 2]). In recent years, by using the way of weight functions, a number of extensions of (1) were given by Yang (cf. [3]). Noticing that inequality (1) is with a homogenous kernel of degree −1, a survey of the study of Hilbert-type inequalities with the homogeneous kernels of degree negative numbers and some parameters was given by [4] in 2009. Recently, some inequalities with the homogenous kernels and nonhomogenous kernels have been studied (cf. [5–12]). All of the above integral inequalities are built in the quarter plane of the first quadrant.

In 2007, Yang [13] first gave a Hilbert-type integral inequality with the nonhomogeneous kernel in the whole plane as follows:

(2) ∬−∞∞f(x)g(y)(1+ex+y)λdx dy <B(λ2,λ2)(∫−∞∞e−λxf2(x)dx∫−∞∞e−λyg2(y)dy)1/2,

where the constant factor *B*(*λ*/2, *λ*/2)  (*λ* > 0) is the best possible. If 0 < *λ* < 1, *p* > 1, and (1/*p*) + (1/*q*) = 1, Yang [14] gave another new Hilbert-type integral inequality in the whole plane in 2008 as follows:

(3) ∬−∞∞1|1+xy|λf(x)g(y)dx dy   <kλ{∫−∞∞|x|p(1−(λ/2))−1fp(x)dx}1/p   ×{∫−∞∞|y|q(1−(λ/2))−1gq(y)dy}1/q,

where the constant factor

(4) $$\begin{array}{c} k_{\lambda }:=B\left(\frac{\lambda }{2},\frac{\lambda }{2}\right)+2B\left(1-\lambda ,\frac{\lambda }{2}\right) \end{array}$$

is the best possible. He et al. [15–20] also provided some Hilbert-type integral inequalities in the whole plane by using some new methods and techniques.

In this paper, by the use of the way of real analysis, we estimate the weight functions and give some new Hilbert-type integral inequalities in the whole plane with nonhomogeneous kernels and multiparameters, which are extensions of (3). The constant factors related to the hypergeometric function and the beta function are proved to be the best possible. We also consider the equivalent forms, the reverses, and some particular inequalities with the homogeneous kernels.

## 2. Some Lemmas

Assuming that *α* > 0, we have Γ(*α*) = ∫_0_^*∞*^ *x* ^*α*−1^ *e* ^−*x*^ *dx*, where Γ(*α*) is the Γ function (cf. [21]). For *β* > −1, *η* > 0, setting *v* = −*η* ln⁡⁡*t*, we find the following expression:

(5) $$\begin{array}{c} \overset{1}{\underset{0}{\int }}{\left(-\ln t\right)}^{\beta }t^{\eta -1}dt=\frac{1}{\eta^{\beta +1}}=\overset{\infty }{\underset{0}{\int }}v^{(\beta +1)-1}e^{-v}dv=\frac{\Gamma \left(\beta +1\right)}{\eta^{\beta +1}}. \end{array}$$

**Lemma 1 .**

If *β* > −1, min⁡⁡{*μ*, *σ*} > −*α*, *μ* + *σ* = *λ* < 1 + *β*, and *δ* ∈ {−1,1}, define the weight functions *ω*_*δ*_(*σ*, *y*) and *ϖ*_*δ*_(*σ*, *x*) as follows:

(6) ωδ(σ,y):=∫−∞∞|ln⁡|xδy||β|1+xδy|λ    ×(min⁡⁡{1,|xδy|}max⁡⁡{1,|xδy|})α|y|σ|x|1−δσdx,ϖδ(σ,x)≔∫−∞∞|ln⁡|xδy||β|1+xδy|λ    ×(min⁡⁡{1,|xδy|}max⁡⁡{1,|xδy|})α|x|δσ|y|1−σdy.

Then, for *y*, *x* ∈ (−*∞*, 0)∪(0, +*∞*), we have

(7) ωδ(σ,y)=ϖδ(σ,x)=Kβ(σ)≔2Γ(β+1)∑k=0∞(λ+2k−12k) ×[1(2k+σ+α)β+1    +1(2k+μ+α)β+1]∈R+.

**Proof**

(i) For *δ* = 1, setting *u* = *xy*, we find, for *y* ∈ (−*∞*, 0)∪(0, +*∞*),

(8) ω1(σ,y)=∫−∞∞|ln⁡⁡|u||β|1+u|λ(min⁡{1,|u|}max⁡{1,|u|})α|u|σ−1du  =∫0∞|ln⁡⁡u|β(min⁡⁡{1,u})αuσ−1(1+u)λ(max⁡{1,u})αdu   +∫−∞0|ln⁡⁡(−u)|β(min⁡⁡{1,−u})α|1+u|λ(max⁡⁡{1,−u})α(−u)σ−1du  =∫0∞[|ln⁡⁡u|β(min⁡⁡{1,u})αuσ−1(1+u)λ(max⁡⁡{1,u})α      +|ln⁡⁡u|β(min⁡⁡{1,u})αuσ−1|1−u|λ(max⁡⁡{1,u})α]du  =∫01[(−ln⁡⁡u)βuσ+α−1(1+u)λ+(−ln⁡⁡u)βuσ+α−1(1−u)λ]du   +∫1∞[(ln⁡⁡u)βuσ−α−1(1+u)λ+(ln⁡⁡u)βuσ−α−1(u−1)λ]du.

By Lebesgue term-by-term integration theorem (cf. [22]), in view of (8) and (5), we find

(9) ω1(σ,y)=∫01[1(1+u)λ+1(1−u)λ]   ×(−ln⁡u)β(uσ+α−1+uμ+α−1)du=∫01∑k=0∞(−λk)[uk+(−u)k]   ×(−ln⁡u)β(uσ+α−1+uμ+α−1)du=2∫01∑k=0∞(λ+2k−12k)    ×(−ln⁡u)β(u2k+σ+α−1+u2k+μ+α−1)du=2∑k=0∞(λ+2k−12k)    ×∫01(−ln⁡u)β(u2k+σ+α−1+u2k+μ+α−1)du=2Γ(β+1)∑k=0∞(λ+2k−12k) ×[1(2k+σ+α)β+1+1(2k+μ+α)β+1].

(ii) For *δ* = −1, setting *y*/*x*, we still can obtain *ω*_−1_(*σ*, *y*) = *K*_*β*_(*σ*). Setting *u* = *x* ^*δ*^ *y*, we find

(10) $$\begin{array}{c} \varpi_{\delta }\left(\sigma ,x\right)=\overset{\infty }{\underset{-\infty }{\int }}\frac{{\left|\ln\left|u\right|\right|}^{\beta }}{{\left|1+u\right|}^{\lambda }}{\left(\frac{\min\left\{1,\left|u\right|\right\}}{\max\left\{1,\left|u\right|\right\}}\right)}^{\alpha }{\left|u\right|}^{\sigma -1}du=K_{\beta }\left(\sigma \right). \end{array}$$

Since, for *β* > −1,  0 < *θ*_0_ < min⁡{*μ* + *α*, *σ* + *α*},

(11) (−ln⁡u1−u)βuθ0⟶0 (u⟶0+),(−ln⁡u1−u)βuθ0⟶1 (u⟶1−),

there exists a positive number *L*, such that ((−ln⁡*u*)/(1 − *u*))^*β*^ *u* ^*θ*_0_^ ≤ *L*(*u* ∈ (0,1]);   then, by (9), it follows that

(12) 0<Kβ(σ)≤2L∫01uσ+α−θ0−1+uμ+α−θ0−1(1−u)λ−βdu=2L[B(1−λ+β,σ+α−θ0)    +B(1−λ+β,μ+α−θ0)]<∞,

and then *K*_*β*_(*σ*) ∈ **R**_+_. Hence we have (7).

**Remark 2 .**

We have the following formula of the hypergeometric function *F* (cf. [21]). If *Re*(*γ*) > *Re*(*θ*) > 0, |arg(1 − *z*)|<*π*, then

(13) F(α,θ,γ,z):=Γ(γ)Γ(θ)Γ(γ−θ) ×∫01tθ−1(1−t)γ−θ−1(1−zt)−αdt.

In particular, for *z* = −1, *γ* = *θ* + 1  (*θ* > 0), it follows that

(14) $$\begin{array}{c} \overset{1}{\underset{0}{\int }}t^{\theta -1}{\left(1+t\right)}^{-\alpha }dt=\frac{1}{\theta }F\left(\alpha ,\theta ,1+\theta ,-1\right)\in R_{+}. \end{array}$$

In (9), for *β* = 0  (*λ* < 1), in view of (14), we have

(15) K0(σ)=∫01[1(1+u)λ+1(1−u)λ]   ×(uσ+α−1+uμ+α−1)du=1σ+αF(λ,σ+α,1+σ+α,−1) +1μ+αF(λ,μ+α,1+μ+α,−1) +B(1−λ,σ+α)+B(1−λ,μ+α)∈R+.

**Lemma 3 .**

If *p* > 1,  (1/*p*)+(1/*q*) = 1, *β* > −1,  min⁡{*μ*, *σ*} > −*α*, *μ* + *σ* = *λ* < 1 + *β*, *δ* ∈ {−1,1}, *K*_*β*_(*σ*) is indicated by (7), and *f*(*x*) is a nonnegative measurable function in (−*∞*, *∞*), then one has

(16) J≔∫−∞∞|y|pσ−1[∫−∞∞|ln⁡|xδy||β|1+xδy|λ            ×(min⁡{1,|xδy|}max⁡{1,|xδy|})αf(x)dx]pdy≤Kβp(σ)∫−∞∞|x|p(1−δσ)−1fp(x)dx.

**Proof**

By Hölder's inequality (cf. [23]), we have

(17) [∫−∞∞|ln⁡⁡|xδy||β|1+xδy|λ(min⁡⁡{1,|xδy|}max⁡⁡{1,|xδy|})αf(x)]p =[∫−∞∞|ln⁡⁡|xδy||β|1+xδy|λ(min⁡⁡{1,|xδy|}max⁡⁡{1,|xδy|})α      ×(|x|(1−δσ)/q|y|(1−σ)/qf(x))(|y|(1−σ)/q|x|(1−δσ)/q)dx]p ≤∫−∞∞|ln⁡⁡|xδy||β|1+xδy|λ(min⁡⁡{1,|xδy|}max⁡⁡{1,|xδy|})α     ×|x|(1−δσ)(p−1)|y|1−σfp(x)dx  ×(∫−∞∞|ln⁡⁡|xδy||β|1+xδy|λ(min⁡{1,|xδy|}max⁡{1,|xδy|})α        ×|y|(1−σ)(q−1)|x|1−δσdx)p−1 =(ωδ(σ,y))p−1|y|pσ−1  ×∫−∞∞|ln⁡⁡|xδy||β|1+xδy|λ(min⁡{1,|xδy|}max⁡{1,|xδy|})α      ×|x|(1−δσ)(p−1)|y|1−σfp(x)dx.

Then, by (7) and Fubini theorem (cf. [22]), it follows that

(18) J≤Kβp−1(σ)∫−∞∞[∫−∞∞|ln⁡|xδy||β|1+xδy|λ(min⁡⁡{1,|xδy|}max⁡⁡{1,|xδy|})α            ×|x|(1−δσ)(p−1)|y|1−σfp(x)dx]dy=Kβp−1(σ)∫−∞∞ϖδ(σ,x)|x|p(1−δσ)−1fp(x)dx.

Hence, in view of (7), inequality (16) follows.

## 3. Main Results and Applications

**Theorem 4 .**

If *p* > 1,  (1/*p*)+(1/*q*) = 1,  *β* > −1,  min⁡{*μ*, *σ*}>−*α*,  *μ* + *σ* = *λ* < 1 + *β*, and *δ* ∈ {−1,1},  *f*(*x*),  *g*(*y*) ≥ 0, satisfying 0 < ∫_−*∞*_^*∞*^|*x*|^*p*(1−*δσ*)−1^ *f* ^*p*^(*x*)*dx* < *∞* and 0 < ∫_−*∞*_^*∞*^ | *y*|^*q*(1−*σ*)−1^ *g* ^*q*^(*y*)*dy* < *∞*, then one has

(19) I≔∬−∞∞|ln⁡|xδy||β|1+xδy|λ(min⁡{1,|xδy|}max⁡{1,|xδy|})α     ×f(x)g(y)dx dy<Kβ(σ){∫−∞∞|x|p(1−δσ)−1fp(x)dx}1/p ×{∫−∞∞|y|q(1−σ)−1gq(y)dy}1/q,

(20) J≔∫−∞∞|y|pσ−1[∫−∞∞|ln⁡|xδy||β|1+xδy|λ            ×(min⁡{1,|xδy|}max⁡{1,|xδy|})αf(x)dx]pdy<Kβp(σ)∫−∞∞|x|p(1−δσ)−1fp(x)dx,

where the constant factors *K*_*β*_(*σ*) and *K*_*β*_^*p*^(*σ*) are the best possible and *K*_*β*_(*σ*) is defined by (7). Inequalities (19) and (20) are equivalent.

In particular, for *δ* = 1, we have the following equivalent inequalities:

(21) ∬−∞∞|ln⁡|xy||β|1+xy|λ(min⁡{1,|xy|}max⁡{1,|xy|})α    ×f(x)g(y)dx dy <Kβ(σ){∫−∞∞|x|p(1−σ)−1fp(x)dx}1/p  ×{∫−∞∞|y|q(1−σ)−1gq(y)dy}1/q,∫−∞∞|y|pσ−1[∫−∞∞|ln⁡|xy||β|1+xy|λ(min⁡{1,|xy|}max⁡{1,|xy|})α           ×f(x)dx]pdy <Kβp(σ)∫−∞∞|x|p(1−σ)−1fp(x)dx.

**Proof**

If (17) takes the form of equality for a *y* ∈ (−*∞*, 0)∪(0, *∞*), then there exist constants *A* and *B*, such that they are not all zero, and

(22) A|x|(1−δσ)(p−1)|y|1−σfp(x) =B|y|(1−σ)(q−1)|x|1−δσ  a.e.  in  (−∞,∞).

We suppose that *A* ≠ 0 (otherwise *B* = *A* = 0). Then it follows that

(23) $$\begin{array}{c} {\left|x\right|}^{p(1-\delta \sigma )-1}f^{p}\left(x\right)={\left|y\right|}^{q(1-\sigma )}\frac{B}{A\left|x\right|}\;\;\text{a.e.}\;\;\text{in}\;\;\left(-\infty ,\infty \right), \end{array}$$

which contradicts the fact that 0 < ∫_−*∞*_^*∞*^|*x*|^*p*(1−*δσ*)−1^ *f* ^*p*^(*x*)*dx* < *∞*. Hence (17) takes the form of strict inequality and so does (16). Then we have (20). By Hölder's inequality (cf. [23]), we find

(24) I=∫−∞∞[|y|σ−(1/p)∫−∞∞|ln⁡|xδy||β|1+xδy|λ             ×(min⁡{1,|xδy|}max⁡{1,|xδy|})αf(x)dx] ×(|y|(1/p)−σg(y)dy)≤J1/p{∫−∞∞|y|q(1−σ)−1gq(y)dy}1/q.

By (20), we have (19). On the other hand, suppose that (19) is valid. We set

(25) g(y)≔|y|pσ−1[∫−∞∞|ln⁡|xδy||β|1+xδy|λ         ×(min⁡{1,|xδy|}max⁡{1,|xδy|})αf(x)dx]p−1,

and find *J* = ∫_−*∞*_^*∞*^ | *y*|^*q*(1−*σ*)−1^ *g* ^*q*^(*y*)*dy*. By (16), we have *J* < *∞*. If *J* = 0, then (20) is obviously value; if 0 < *J* < *∞*, then, by (19), we obtain

(26) 0<∫−∞∞|y|q(1−σ)−1gq(y)dy=J=I<Kβ(σ){∫−∞∞|x|p(1−δσ)−1fp(x)dx}1/p ×{∫−∞∞|y|q(1−σ)−1gq(y)dy}1/q<∞,J1/p={∫−∞∞|y|q(1−σ)−1gq(y)dy}1/p<Kβ(σ){∫−∞∞|x|p(1−δσ)−1fp(x)dx}1/p.

Hence we have (20), which is equivalent to (19). We indicate two sets *E*_*δ*_≔{*x* ∈ **R**; |*x*|^*δ*^ ≥ 1} and *E*_*δ*_^+^≔*E*_*δ*_∩**R**_+_ = {*x* ∈ **R**_+_; *x* ^*δ*^ ≥ 1}. For *ɛ* > 0, we define two functions $\tilde{f}(x)$, $\tilde{g}(y)$ as follows:

(27) f~(x)≔{|x|δ(σ−(2ɛ/p))−1,x∈Eδ,0,x∈R∖Eδ,g~(y)≔{0,y∈(−∞,−1)∪(1,∞),|y|σ+(2ɛ/q)−1,y∈[−1,1].

Then we obtain

(28) L~≔{∫−∞∞|x|p(1−δσ)−1f~p(x)dx}1/p ×{∫−∞∞|y|q(1−σ)−1g~q(y)dy}1/q=2{∫Eδ+x−2δɛ−1dx}1/p ×{∫01y2ɛ−1dy}1/q=1ɛ.

Since, for *Y* = −*y*, we find

(29) h(x)≔∫−11|ln⁡|xδy||β|1+xδy|λ(min⁡{1,|xδy|}max⁡{1,|xδy|})α      ×|y|σ+(2ε/q)−1dy  =∫−11|ln⁡|(−x)δY||β|1+(−x)δY|λ(min⁡{1,|(−x)δY|}max⁡{1,|(−x)δY|})α      ×|Y|σ+(2ε/q)−1dY=h(−x),

and *h*(*x*) is an even function, then it follows that

(30) I~≔∬−∞∞|ln⁡|xδy||β|1+xδy|λ(min⁡{1,|xδy|}max⁡{1,|xδy|})α     ×f~(x)g~(y)dx dy=∫Eδ|x|δ(σ−(2ε/p))−1h(x)dx=2∫Eδ+xδ(σ−(2ε/p))−1h(x)dx=u=xδy2∫Eδ+x−2δε−1      ×[∫−xδxδ|ln⁡|u||β(min⁡{1,|u|})α|u|σ+(2ε/q)−1|1+u|λ(max⁡{1,|u|})αdu]dx.

Setting *v* = *x* ^*δ*^ in the above integral, by Fubini theorem (cf. [22]), we find

(31) I~=v=xδ2∫1∞v−2ɛ−1     ×[∫−vv|ln⁡|u||β(min⁡⁡{1,|u|})α|u|σ+(2ɛ/q)−1|1+u|λ(max⁡{1,|u|})αdu]dv=2∫1∞v−2ɛ−1{∫0v[|ln⁡u|β(min⁡{1,u})α|1−u|λ(max⁡{1,u})α           +|ln⁡u|β(min⁡{1,u})α(1+u)λ(max⁡{1,u})α]         ×uσ+(2ɛ/q)−1du}dv=2∫1∞v−2ɛ−1    ×{∫01[(−ln⁡u)β(1−u)λ+(−ln⁡u)β(1+u)λ]uσ+α+(2ɛ/q)−1du}dv +2∫1∞v−2ɛ−1     ×{∫1v[(ln⁡u)β(u−1)λ+(ln⁡u)β(1+u)λ]uσ−α+(2ɛ/q)−1du}dv=1ɛ∫01[(−ln⁡u)β(1−u)λ+(−ln⁡u)β(1+u)λ]uσ+α+(2ɛ/q)−1du +2∫1∞(∫u∞v−2ɛ−1dv)      ×[(ln⁡u)β(u−1)λ+(ln⁡u)β(1+u)λ]uσ−α+(2ɛ/q)−1du=1ɛ{∫01[(−ln⁡u)β(1−u)λ+(−ln⁡u)β(1+u)λ]uσ+α+(2ɛ/q)−1du    +∫1∞[(ln⁡u)β(u−1)λ+(ln⁡u)β(1+u)λ]uσ−α−(2ɛ/p)−1du}.

If the constant factor *K*_*β*_(*σ*) in (19) is not the best possible, then there exists a positive number *k* with *K*_*β*_(*σ*) < *k*, such that (19) is valid when replacing *K*_*β*_(*σ*) by *k*. Then we have $\tilde{I}<k\tilde{L}$, and

(32) ∫01[(−ln⁡u)β(1−u)λ+(−ln⁡u)β(1+u)λ]uσ+α+(2ɛ/q)−1du  +∫1∞[(ln⁡u)β(u−1)λ+(ln⁡u)β(1+u)λ]uσ−α−(2ɛ/p)−1du =ɛI~<ɛkL~=k.

By (8) and Fatou lemma (cf. [22]), we have

(33) Kβ(σ)=∫01[(−ln⁡u)β(1−u)λ+(−ln⁡u)β(1+u)λ]uσ+α−1du +∫1∞[(ln⁡u)β(u−1)λ+(ln⁡u)β(1+u)λ]uσ−α−1du=∫01lim⁡ɛ→0+[(−ln⁡u)β(1−u)λ+(−ln⁡u)β(1+u)λ]uσ+α+(2ɛ/q)−1du +∫1∞lim⁡ɛ→0+[(ln⁡u)β(u−1)λ+(ln⁡u)β(1+u)λ]uσ−α−(2ɛ/p)−1du≤lim⁡_ɛ→0+{∫01[(−ln⁡u)β(1−u)λ+(−ln⁡u)β(1+u)λ]uσ+α+(2ɛ/q)−1du      +∫1∞[(ln⁡u)β(u−1)λ+(ln⁡u)β(1+u)λ]         ×uσ−α−(2ɛ/p)−1du}≤k,

which contradicts the fact that *k* < *K*_*β*_(*σ*). Hence the constant factor *K*_*β*_(*σ*) in (19) is the best possible. If the constant factor in (20) is not the best possible, then, by (24), we may get a contradiction that the constant factor in (19) is not the best possible.

**Theorem 5 .**

As the assumptions of Theorem 4, replacing *p* > 1 by 0 < *p* < 1, one has the equivalent reverses of (19) and (20) with the same best constant factors.

**Proof**

By the reverse Hölder's inequality (cf. [23]), we have the reverses of (16) and (24). It is easy to obtain the reverse of (20). In view of the reverses of (20) and (24), we obtain the reverse of (19). On the other hand, suppose that the reverse of (19) is valid. Setting the same *g*(*y*) as (25) in Theorem 4, by the reverse of (16), we have *J* > 0. If *J* = *∞*, then the reverse of (20) is obviously value; if *J* < *∞*, then, by the reverse of (19), we obtain the reverses of (26). Hence we have the reverse of (20), which is equivalent to the reverse of (19). If the constant factor *K*_*β*_(*σ*) in the reverse of (19) is not the best possible, then there exists a positive constant *k*, with *k* > *K*_*β*_(*σ*), such that the reverse of (19) is still valid when replacing *K*_*β*_(*σ*) by *k*. By the reverse of (32), we have

(34) ∫01[(−ln⁡u)β(1−u)λ+(−ln⁡u)β(1+u)λ]uσ+α+(2ɛ/q)−1du +∫1∞[(ln⁡u)β(u−1)λ+(ln⁡u)β(1+u)λ]uσ−α−(2ɛ/p)−1du>k.

For *ɛ* → 0^+^, by Levi theorem (cf. [22]), we find

(35) ∫1∞[(ln⁡u)β(u−1)λ+(ln⁡u)β(1+u)λ]uσ−α−(2ɛ/p)−1du ⟶∫1∞[(ln⁡u)β(u−1)λ+(ln⁡u)β(1+u)λ]uσ−α−1du.

There exists a constant *δ*_0_ > 0, such that *σ* − (1/2)*δ*_0_ > −*α*, and then 0 < *K*_*β*_(*σ* − (*δ*_0_/2)) < *∞*. For 0 < *ɛ* < *δ*_0_|*q*|/4  (*q* < 0), since *u* ^*σ*+*α*+(2*ɛ*/*q*)−1^ ≤ *u* ^*σ*+*α*−(*δ*_0_/2)−1^, *u* ∈ (0,1], and

(36) $$\begin{array}{c} \overset{1}{\underset{0}{\int }}\left[\frac{{\left(-\ln u\right)}^{\beta }}{{\left(1-u\right)}^{\lambda }}+\frac{{\left(-\ln u\right)}^{\beta }}{{\left(1+u\right)}^{\lambda }}\right]u^{\sigma +\alpha -(\delta_{0}/2)-1}du\leq K_{\beta }\left(\sigma -\frac{\delta_{0}}{2}\right), \end{array}$$

then, by Lebesgue control convergence theorem (cf. [22]), for *ɛ* → 0^+^, we have

(37) ∫01[(−ln⁡u)β(1−u)λ+(−ln⁡u)β(1+u)λ]uσ+α+(2ɛ/q)−1du ⟶∫01[(−ln⁡u)β(1−u)λ+(−ln⁡u)β(1+u)λ]uσ+α−1du.

By (34), (35), and (37), for *ɛ* → 0^+^, we have *K*_*β*_(*σ*) ≥ *k*, which contradicts the fact that *k* > *K*_*β*_(*σ*). Hence, the constant factor *K*_*β*_(*σ*) in the reverse of (19) is the best possible. If the constant factor in the reverse of (20) is not the best possible, then, by the reverse of (24), we may get a contradiction that the constant factor in the reverse of (19) is not the best possible.

**Remark 6 .**

(i) For *δ* = −1 in (19) and (20), replacing |*x*|^*λ*^ *f*(*x*) by *f*(*x*), we obtain the following equivalent inequalities with a homogeneous kernel and the best possible constant factors:

(38) ∬−∞∞|ln⁡|y/x||β|x+y|λ(min⁡{|x|,|y|}max⁡{|x|,|y|})αf(x)g(y)dx dy <Kβ(σ){∫−∞∞|x|p(1−μ)−1fp(x)dx}1/p  ×{∫−∞∞|y|q(1−σ)−1gq(y)dy}1/q,

(39) ∫−∞∞|y|pσ−1  ×[∫−∞∞|ln⁡|y/x||β|x+y|λ(min⁡{|x|,|y|}max⁡{|x|,|y|})αf(x)dx]pdy <Kβp(σ)∫−∞∞|x|p(1−μ)−1fp(x)dx.

(ii) For *β* = 0  (*λ* < 1) in (19) and (20), we obtain the following equivalent inequalities:

(40) ∬−∞∞(min⁡⁡{1,|xδy|})α|1+xδy|λ(max⁡{1,|xδy|})αf(x)g(y)dx dy <K0(σ){∫−∞∞|x|p(1−δσ)−1fp(x)dx}1/p  ×{∫−∞∞|y|q(1−σ)−1gq(y)dy}1/q,

(41) ∫−∞∞|y|pσ−1   ×[∫−∞∞(min⁡{1,|xδy|})α|1+xδy|λ(max⁡{1,|xδy|})αf(x)dx]pdy <K0p(σ)∫−∞∞|x|p(1−δσ)−1fp(x)dx,

where *K*_0_(*σ*) is indicated by (15).

(iii) For *α* = 0,  *σ* = *μ* = *λ*/2  (0 < *λ* < 1) in (40), we find

(42) K0(λ2)=∫−∞∞1|1+u|λ|u|(λ/2)−1du=∫0∞u(λ/2)−1du(1+u)λ+∫−∞−1(−u)(λ/2)−1du(−1−u)λ +∫−10(−u)(λ/2)−1du(1+u)λ=B(λ2,λ2)+2∫01u(λ/2)−1(1−u)λdu=kλ,

and then (3) follows. Hence, (40) and (19) are extensions of (3).
